# Influence of the RNase H domain of retroviral reverse transcriptases on the metal specificity and substrate selection of their polymerase domains

**DOI:** 10.1186/1743-422X-6-159

**Published:** 2009-10-08

**Authors:** Tanaji T Talele, Alok Upadhyay, Virendra N Pandey

**Affiliations:** 1 Department of Biochemistry and Molecular Biology, UMDNJ-New Jersey Medical School, 185 South Orange Avenue, Newark, NJ 07103, USA; 2 Department of Pharmaceutical Sciences, College of Pharmacy and Allied Health Professions, St John's University, 8000 Utopia Parkway, Jamaica, NY 11439, USA

## Abstract

Reverse transcriptases from HIV-1 and MuLV respectively prefer Mg^2+ ^and Mn^2+ ^for their polymerase activity, with variable fidelity, on both RNA and DNA templates. The function of the RNase H domain with respect to these parameters is not yet understood. To evaluate this function, two chimeric enzymes were constructed by swapping the RNase H domains between HIV-1 RT and MuLV RT. Chimeric HIV-1 RT, having the RNase H domain of MuLV RT, inherited the divalent cation preference characteristic of MuLV RT on the DNA template with no significant change on the RNA template. Chimeric MuLV RT, likewise partially inherited the metal ion preference of HIV-1 RT. Unlike the wild-type MuLV RT, chimeric MuLV RT is able to use both Mn.dNTP and Mg.dNTP on the RNA template with similar efficiency, while a 30-fold higher preference for Mn.dNTP was seen on the DNA template. The metal preferences for the RNase H activity of chimeric HIV-1 RT and chimeric MuLV RT were, respectively, Mn^2+ ^and Mg^2+^, a property acquired through their swapped RNase H domains. Chimeric HIV-1 RT displayed higher fidelity and discrimination against rNTPs than against dNTPs substrates, a property inherited from MuLV RT. The overall fidelity of the chimeric MuLV RT was decreased in comparison to the parental MuLV RT, suggesting that the RNase H domain profoundly influences the function of the polymerase domain.

## Introduction

Retroviral reverse transcriptases (RTs) are responsible for copying the viral genomic RNA into double-stranded DNA by a multi-step reverse transcription process. A constituent of the pol gene, RT is proteolytically processed from the gag-pol polyprotein precursor [1,2]. The subunit organization of mature RTs from various viruses is different. Reverse transcriptase from MMTV and MuLV [3,4] are monomers, whereas those from HIV-1, HIV-2, SIV, FIV, EIAV, and AMV are heterodimers. This enzyme is multifunctional, exhibiting both RNA- and DNA-dependent polymerase activities, as well as an RNase H activity that is both polymerase-dependent and polymerase-independent [1,5-8]. Based on the amino acid sequence alignment of the various reverse transcriptases and other polymerases, it has been proposed that the DNA polymerase activity resides in the N-terminal domain, whereas the C-terminal harbors the RNase H activity [9,10]. These domain assignments are supported by mutational studies [4] and confirmed by the availability of the 3-dimensional crystal structure of HIV-1 RT [11,12]. Considerable homology exists between the RNase H domains of retroviral RTs and the *E. coli *RNase H [13-16]. In a model of the MuLV RT RNase H domain based on the structure of *E. coli *RNase H [13], the position of the active site residues, D524, E562, D583, and D653, is similar to the position of residues D443, E478, D498, and D549 in the crystal structure of HIV-1 RT RNase H [14], thus suggesting that they share structural similarities.

There are two metal binding sites in the crystal structure of HIV-1 RT RNase H, whereas only a single metal binding site has been reported in *E. coli *RNase H [16]. However, the co-crystal structure of *E. coli *RNase H with Mn^2+ ^also shows two distinct metal binding sites [17]. HIV-1 RT catalyzes the double-stranded RNA cleavage in the presence of Mn^2+^, while no such activity is seen with Mg^2+^, suggesting distinct sites for these two metals [18]. This finding has been supported by mutational studies. A point mutation in the RNase H domain of HIV-1 RT substituting Glu→Gln at the 478 position renders the enzyme inactive with Mg^2+^, but retains Mn^2+^-dependent endoribonuclease and double-stranded RNA cleavage (RNase H*) activities [19].

As with HIV-1 RT, double- stranded RNA cleavage activity of MuLV RT requires the presence of Mn^2+ ^[20], although both enzymes exhibit a distinct metal preference for their polymerase and RNase H activities [21,22]. While MuLV RT prefers Mn^2+ ^as the divalent cation for both of these activities, HIV-1 RT prefers Mg^2+ ^for its polymerase reaction. However, Mn^2+ ^is also used, albeit with lower efficiency [23,24]. In the RNase H domain of HIV-1 RT, Asp 443, Glu 478, and Asp 498 constitute the metal coordinating catalytic triad [14]. It has been suggested that the fourth highly conserved residue, Asp 549, makes an important contribution to RNase H activity, although it is not absolutely required for metal coordination [24-26]. Structural and biochemical studies have demonstrated that Asp 110, Asp 185, and Asp 186 constitute the metal coordinating triad in the polymerase domain of HIV-1 RT [11,12,27-29], while Asp 150, Asp 224, and Asp 225 form the equivalent triad in MuLV RT [30,31].

The two domains of MuLV RT have been shown to be independent of each other [4,32,33], in contrast to HIV-1 RT [25,34-37]. Earlier, we demonstrated that the polymerase domain (p51) of HIV-1 RT lacking polymerase activity can be converted to an active monomeric enzyme when fused with the RNase H domain of MuLV RT [38]. This observation confirms the functional dependence of the polymerase domain of HIV-1 RT on the RNase H domain. Neither the degree of functional interdependence of these domains for their enzymatic activities nor the precise nature of their effect on catalytic function is clear. To explore the subtle influence of the RNase H domain on the biochemical characteristics of the enzyme, we constructed two chimeric enzymes of HIV-1 RT and MuLV-RT by swapping the RNase H domains between them. We observed that the metal preference for the polymerase activity of chimeric HIV-1 RT changed from Mg^2+ ^to Mn^2+^, a property inherited from MuLV RT via its RNase H domain. Here we provide evidence that the metal preference, as well as substrate specificity for the polymerase function of the chimeric RTs, is influenced by the RNase H domains.

## Materials and methods

### Materials

DNA restriction enzymes, DNA modifying enzymes, and dNTP solutions were purchased from Roche Molecular Biochemicals. Fast-flow chelating Sepharose (iminodiacetic Sepharose) for immobilized metal affinity chromatography (IMAC) was purchased from Amersham Pharmacia Biotech, ^32^P-labeled dNTPs and ATP were the products of NEN. The RNA and DNA oligomers used as template primers were synthesized at the Molecular Resource Facility at UMDNJ and have the same sequence as described before [38]. Other reagents, all were of the highest available purity grade, were purchased from Fisher, Millipore Corp., Roche Molecular Biochemicals, and Bio-Rad.

### Construction and Expression of Chimeric Enzymes

Our group has previously described the construction of chimeric HIV-1 RT containing the polymerase domain of HIV-1 RT and the RNase H domain from MuLV RT [38]. The chimeric MuLV RT, having the polymerase domain of MuLV RT and the RNase H domain from HIV-1 RT, was constructed using pET28a-MRT [39] and pKK-RT66 [40-42]; these were the respective sources of the complete coding sequence of MuLV RT and HIV RT. The polymerase domain of MuLV RT, starting from 1 bp-1,560 bp was PCR-amplified using the upstream primer (5' TAT GGG GCC ATA TGA ATA TAG AAG ATG AG 3') and the downstream primer (5' TGG CGA GCT CTA CGT ACC AGG TGG GGT CGG CGT 3'), and pET28aMRT as a template. The upstream and downstream primers respectively contained the unique restriction sites *Nde1 *and *Sac1*. The PCR amplified fragment was digested with *Nde*I and *Sac*I, and cloned at the compatible ends in pET28a. The resulting plasmid (pET28aMPol) was expressed in *E. coli *as the polymerase domain for MuLV RT (M-Pol). Similarly, the RNase H domain of HIV-1 RT starting from 1,324 bp-1,680 bp was PCR-amplified using the upstream primer (5'-CCC AGA CGC CGA CAC CTG GTA GGT AGA TGG GGC AGC TAA CAG G-3'), and the downstream primer (5'-TAT AGG GAC CCT CGA GTA GTA CTT TCC TGA TTC CAG C3'), and pKKRT66 as the template. This PCR-amplified fragment was subcloned at the *SnaBI *and *XhoI *sites of pET28a-M-POL. The recombinant plasmid thus obtained, pET28a-MHCI, was expressed in *E. coli *BL21 (DE3) pLysS as MHCI RT.

### Glycerol gradient ultracentrifugation

Fifty micrograms of each enzyme protein in Tris - NaCl buffer (50 mM Tris HCl, pH 8.0, and 400 mM NaCl) was loaded onto 5 ml of 10%-30% linear glycerol gradient prepared in the same buffer [38]. Gradients were centrifuged at 48,000 rpm for 20-24 h in a SW 50.1 rotor. Gradients were fractionated from the bottom and subjected to SDS-polyacrylamide gel electrophoresis to determine the protein peak fraction.

### Polymerase Assay

The activity of the wild-type and chimeric enzymes was determined using the homopolymeric template primer poly (rA). (dT)_18 _and the heteropolymeric U5-PBS HIV-1 RNA, with DNA templates primed with 17-mer PBS primer as described before [42]. In brief, 50 μl of the reaction mixture contained 50 mM Tris-HCl (pH 7.8); 100 μg/ml bovine serum albumin; 2 mM MgCl_2 _or 0.5 mM MnCl_2_; 1 mM dithiothreitol; 60 mM KCl; 100 nM template primer;100-500 μM of all four dNTPs (or TTP alone with homopolymeric rA.dT); 0.5 μCi of α-^32^P-labeled TTP or 0.5 μCi each of α-^32^P-labeled TTP; dGTP per reaction for heteropolymeric templates; and 15-25 nM of the enzyme. Reactions were done at 37°C for the desired time and terminated by the addition of ice cold 5% trichloroacetic acid containing 5 mM inorganic pyrophosphate. The acid-insoluble materials were filtered on Whatman GF/B filters, dried, and counted for radioactivity in a liquid scintillation counter.

### RNase H Activity Assay

We used a 5'-^32^P labeled 30-mer synthetic U5-PBS RNA template annealed with a complementary 30-mer DNA to determine the RNase H activities of the enzymes [38]. The reaction mixture contained labeled RNA-DNA hybrid (20 K cpm); 60 mM KCl; 5 mM MgCl_2 _or 0.5 mM MnCl_2_; 10 mM dithiothreitol; 50 mM Tris-HCl, pH 8.0; 0.1 mg/ml bovine serum albumin; and 100 ng of enzyme in a final volume of 5 μL. Reactions were done at 37°C for variable times and terminated by the addition of equal volumes of Sanger's gel loading dye [43]. The cleavage products were analyzed on an 8% denaturing polyacrylamide-urea gel and scanned on a phosphorImager (Molecular Dynamics).

### Steady-State Kinetic Assays

Kinetic parameters in the presence of Mg^2+ ^or Mn^2+ ^were determined using heteropolymeric RNA and DNA templates as described [42,44,45], except that reactions were done at 37°C instead of room temperature. The concentration of metal ions used was 2 mM Mg^2+^; 0.5 mM Mn^2+^. K_m _and k_cat _values were determined from the Eadie- Hoftsee plots using the enzyme kinetic program.

### Gel Shift Assay

The K_d _values for template-primer (DNA-DNA) binding to the wild- type enzymes and their chimeric derivatives were determined by gel mobility shift assay using ^32 ^P-labeled 17-mer PBS primer annealed with the 49-mer DNA template. The labeled template-primer was present at a final concentration of 5 nM in a total reaction volume of 10 μL containing 50 mM Tris-HCl (pH 7.8), 60 mM KCl, 1 mM DTT, 0.01% NP40, 10% glycerol, and varying concentrations of enzyme proteins. Samples were loaded on a 6% nondenaturing polyacrylamide gel in Tris-borate buffer, pH 8.2. The gel was run at 150 V at 4°C, dried, and subjected to phosphorimaging. The enzyme-DNA binary complex was quantitated using Image Quant software (Molecular Dynamics). The fraction of bound DNA was plotted against the enzyme concentration and the Kd value was obtained as the RT concentration at which 50% of the DNA was bound.

### Gel Analysis of Primer Extension Products in the Presence ofrNTP Substrates

The ability of the wild-type enzymes and their chimeric derivatives to extend the primer by incorporating ribonucleotides was assessed on both U5-PBS RNA and U5-PBS DNA templates primed with 5'-^32^P-labeled PBS DNA primer as described [44-46]. Reactions were initiated by the addition of 500 μM of Mg.rNTP in a final reaction volume of 5 μL. For comparison, control reactions were also done in the presence of dNTP substrates. The reaction mixtures were incubated at 37°C for 10-30 min and terminated by the addition of an equal volume of Sanger's gel loading dye. The reaction products were resolved by denaturing 12% polyacrylamide-8M urea gel electrophoresis and subjected to phosphorimaging.

### Extension of Primers in the Presence of Three dNTPs

5'-^32^P-labeled 17-mer primer annealed with a 2-fold molar excess of 49-mer U5-PBS HIV-1 DNA template was used to assess the fidelity of nucleotide incorporation under conditions in which the biased dNTP pools containing only three dNTPs were supplied [47]. The labeled template primer was incubated with the enzymes at 37°C for 30 min in a total volume of 5 μl containing 50 mM Tris-HCl (pH 7.5), 1 mM DTT, 0.1 mg/ml BSA, 2 mM MgCl_2 _and only 3 dNTPs, each at a 100-μM concentration. The dNTPs used were of the highest available purity grade (HPLC purified) and supplied as 0.1 M solution (Boehringer Mannheim). At the end of incubation, the reaction was quenched by the addition of 5 μl of stop solution containing 40 mM EDTA, 0.014% each of bromophenol blue and xylene cyanol, and 85% formamide. The reaction products were analyzed on a denaturing 8% polyacrylamide-8 M urea gel and visualized on a phosphorimager.

## Results

### Construction, Expression, and Purification of the ChimericEnzymes

**Figure 1:**
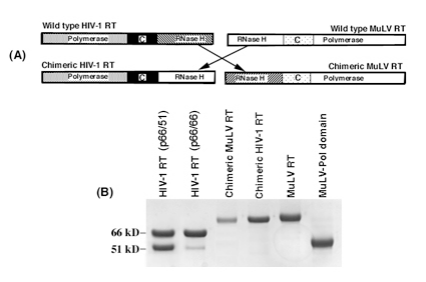
**(A) Schematic representation showing the polymerase connection and RNase H domains of wild-type RTs and their chimeric derivatives**. Swapping of the RNase H domain between the wild-type HIV-1 RT and MuLV RT to construct their chimeric derivatives is shown by arrows. **(B) Coomassie Blue stained SDS polyacrylamide gel of the wild-type enzymes and their chimeric derivatives**. An aliquot of purified chimeric enzymes, M-pol, wild-type p66/66 HIV-1 RT, and MuLV RT was resolved by SDS-PAGE; protein bands were visualized by Coomassie blue staining. In the wild-type HIV-1 RT lane, the minor band seen at the 51 kD position may have been generated by proteolytic cleavage during purification. The positions corresponding to 66 kD and 51 kD are indicated on the left

The chimeric HIV-1 RT and MuLV RT were constructed by swapping the RNase H domain between the reverse transcriptases from HIV-1 and MuLV (Figure 1A). The wild-type enzymes and their chimeric derivatives were expressed in *E. coli *and purified to homogeneity. The chimeric enzymes were in the soluble fraction of the cell extract. Their expression and gel electrophoresis patterns were similar to those of the wild-type enzyme, indicating that there was no deleterious change in their global conformation. The purity of the enzyme preparations was greater than 95%. An SDS-polyacrylamide gel of purified enzymes stained with Coomassie blue is shown in Figure 1B. The enzyme stocks were stored at -70°C for several months without any significant change in polymerase activity.

### Dimeric/Monomeric Conformation of the Chimeric Enzymes

**Figure 2:**
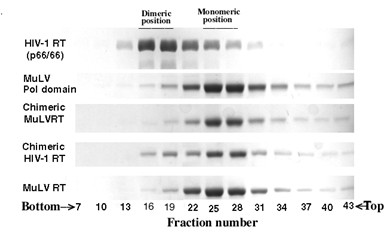
**Glycerol gradient ultracentrifugation analyses of the wild-type enzymes and their chimeric derivatives**. The enzyme proteins were individually resolved by glycerol gradient ultracentrifugation analysis as described. Gradients were fractionated from the bottom and subjected to SDS polyacrylamide gel electrophoresis followed by Coomassie blue staining.

Earlier, we showed that the chimeric HIV-1 RT containing the native DNA polymerase domain from HIV-1 RT and the exotic RNase H domain from MuLV RT is functionally active in the monomeric conformation [38]. To determine the subunit organization of the chimeric MuLV RT containing the exotic RNase H domain from HIV-1 RT, we therefore performed sedimentation analysis of the chimeric MuLV RT, along with the chimeric HIV-1 RT, and their wild-type parental enzymes [38]. The fractions were collected from the bottom and an aliquot of each fraction was analyzed by SDS polyacrylamide gel electrophoresis followed by Coomassie blue staining. Both the chimeric RTs, as well as monomeric MuLV RT, sedimented as monomeric proteins between fractions 25-29, whereas the dimeric HIV-1 RT sedimented at the bottom of the gradient, between fractions 17-21 (Figure 2). This sedimentation profile of the chimeric RTs clearly suggests their monomeric status.

### Metal Preference for the Polymerase Activity Catalyzed by the Wild-Type Enzymes and Their Chimeric Derivatives

The RNA-dependent DNA polymerase activity of the chimeric enzymes was examined using the homopolymeric poly (rA). (dT)_18 _and the heteropolymeric U5-PBS RNA transcript primed with the 17-mer PBS DNA primer. The percentage activity of these enzymes with respect to the wild-type enzyme at 500 μM substrate concentration is shown in Table 1. Wild-type HIV-1 RT consistently showed higher DNA polymerase activity on all three templates with Mg^2+ ^as the divalent cation, while the reverse was true with the wild-type MuLV RT. In contrast, chimeric HIV-1 RT containing the RNase H domain from MuLV RT exhibited a preference for Mn^2+ ^with heteropolymeric U5-PBS DNA and homopolymeric RNA templates, but for Mg^2+ ^with a heteropolymeric RNA template. This change in metal preference may be a consequence of the presence of the RNase H domain of MuLV RT. A similar change in metal preference was also observed with chimeric MuLV RT containing the RNase H domain from HIV-1 RT. This enzyme exhibited similar preference for Mg^2+ ^and Mn^2+ ^with DNA template, while retaining a strong preference for Mn^2+ ^with RNA templates. Curiously, M-Pol of MuLV RT (devoid of the RNase H domain) is able to use Mg^2+ ^and Mn^2+ ^to the same extent with heteropolymeric RNA (65%-68%) and DNA templates (48%-52%) while retaining wild- type preference for Mn^2+ ^on the homopolymeric RNA template. The activity profile (Table 1) determined with saturating concentrations of the metal complexed dNTPs (2 mM) may not reflect true metal preference. Therefore, to assess the metal ion preference of the chimeric enzymes, we determined their steady-state kinetic parameters in the presence of different metal ions.

**Table 1:** Polymerase Activity of the Wild-Type and Chimeric Reverse Transcriptase

**Percentage of Wild-Type HIV-1 RT Polymerase Activity**						
**Enzyme**	**Poly** **(rA).(dT)**_18_		**U5-PBS RNA/17mer DNA**		**U5-PBS 49mer DNA/17mer DNA**	
	**Mg^2+^**	**Mn^2+^**	**Mg^2+^**	**Mn^2+^**	**Mg^2+^**	**Mn^2+^**
WT HIV-1 RT	100 (22649)	100 (6182)	100 (4500)	100 (3740)	100 (2721)	100 (2451)
Chimeric HIV-1 RT	24	96	91	69	46	58
WT MuLV RT	130	331	71	95	58	68
Chimeric MuLV RT	80	477	108	183	81	72
MuLV RT- Pol Domain	88	221	65	68	48	52

The polymerase activities of wild-type reverse transcriptase enzymes and their chimeric derivatives were determined on homopolymeric and heteropolymeric template primers in the presence of Mg^2+ ^or Mn^2+ ^as the divalent cation. The values represent the percentage of WT HIV-1 RT activity. Data shown are the average of three independent experiments. The values in parentheses are the total cpm of acid-insoluble dNMP incorporated into the primer DNA by 100 ng of the WT HIV-1 RT at 37°C in 15 min. These determinations were done at saturating substrate concentrations (500 μM of each dNTP).

### Influence of Mg^2+ ^and Mn^2+ ^on Steady-State Kinetic Parameters of the Wild-Type Enzymes and Their Chimeric Derivatives

The change in metal ion preference observed with the chimeric derivatives of HIV-1 RT and MuLV RT suggests that swapping the RNase H domains between these two RTs imparts some of the characteristics of the parental enzyme. Exploring this possibility, we examined the kinetic parameters of the wild-type enzymes and their chimeric derivatives on U5-PBS RNA and DNA templates. As shown in Table 2, the metal ion preference of chimeric HIV-1 RT exhibited earlier (see Table 1) was confirmed by our steady-state kinetic studies. The catalytic efficiency (k_cat_/K_m_) for this chimeric enzyme was approximately two-fold higher with Mn^2+ ^than with Mg^2+ ^on the DNA template and two-fold higher with Mg^2+ ^on the RNA template. Chimeric MuLV RT, on the other hand, exhibited equal catalytic efficiency with both metal ions on the RNA template while retaining the parental preference for Mn^2+ ^on the DNA template. In contrast, M-Pol of MuLV RT retained its parental characteristics, having consistently higher catalytic efficiency with Mn^2+ ^on both RNA and DNA templates. These results are in contrast to those shown in Table 1. A possible explanation for this discrepancy is that the activity assays in Table 1 were done in the presence of saturating concentrations of metal complexed dNTP (2 mM). Under these experimental conditions, the subtle differences in the metal preference noted in the kinetic analysis were abolished.

**Table 2:** Steady State Kinetic Parameters of the Wild Type and Chimeric Reverse Transcriptases

**Template-primer**	**Enzyme**	**K_mdNTP _μM**	**Mn^2+ ^K_cat _S^-1^**	**K_cat_/K_m_** **S^-1 ^M^-1 ^× 10^2^**	**K_mdNTP _μM**	**Mg^2+ ^K_cat _S^-1^**	**K_cat_/K_m_** **S^-1 ^M^-1 ^× 10^2^**
U5-PBS RNA/17-mer DNA	WT HIV-1 RT	5.8	0.009	15.5	1.7	0.005	29.4
	Chim HIV-1 RT	945.5	0.040	0.4	702.8	0.050	0.7
	WT MuLV RT	2.0	0.003	15.0	16.7	0.006	3.6
	Chim MuLV RT	197.6	0.040	2.0	157.0	0.030	1.9
	M-Pol	2.9	0.004	13.8	301.8	0.021	0.7
							
U5-PBS 49-mer DNA/17-mer DNA	WT HIV-1 RT	2.8	0.029	103.6	1.3	0.035	269.2
	Chim HIV-1 RT	204.8	0.024	1.2	691.0	0.048	0.69
	WT MuLV RT	1.6	0.013	81.3	10.6	0.015	14.2
	Chim MuLV RT	14.8	0.055	37.2	433.0	0.048	1.1
	M-Pol	1.4	0.012	85.7	181.5	0.017	0.94

The steady-state kinetic parameters for wild-type reverse transcriptase from HIV-1 and MuLV and their chimeric derivatives were measured on heteropolymeric RNA and DNA template-primer in the presence of Mg^+2 ^and Mn^+2 ^as the divalent cation. These determinations were carried out at subsaturating concentration of dNTP substrates.

This observation suggests that the ability of the chimeric MuLV RT to use Mg.dNTP as efficiently as it does Mn.dNTP on an RNA template may be due to the presence of the RNase H domain of HIV-1 RT. As expected, the wild-type HIV-1 RT and MuLV RT enzymes respectively preferred Mg^2+ ^and Mn^2+ ^as the divalent cation on both RNA and DNA templates, as shown by their catalytic efficiency values. Although, as compared to that of their wild-type parental enzymes, the catalytic efficiencies of the chimeric enzymes were reduced by 2-384-fold (depending on the template used), the subtle changes in their metal preferences appeared to be dictated by the specific RNase H domain in the chimeric enzyme.

### Template Primer Binding Affinity of the Chimeric Enzyme

The lower affinity for dNTPs observed in the chimeric enzymes in the presence of both the metal ions could be due to their altered affinity for the template primer. We therefore determined their template primer binding affinity by gel shift analysis and compared it with those of the parental wild-type enzymes. The results showed no significant difference in the binding affinity of these chimeric enzymes as compared to that of their wild-type counterparts (Table 3). These results suggest that the altered kinetic parameters observed for the dNTP substrates are not related to any change in template-primer binding affinity.

**Table 3:** K_d _Values for Wild-Type Reverse Transcriptases and Their Chimeric Derivatives

**Enzymes**	**Kd (DNA)** **(nM)**
Wild-type HIV-1 RT	3.20
Chimeric HIV-1 RT	1.50
Wild-type MuLV RT	2.70
Chimeric MuLV RT	3.3
M-pol	1.2

The dissociation constant was determined by a mobility shift assay using a hetero-polymeric 49/17-mer template primer. The values represent the average of three independent experiments.

### Use of rNTP versus dNTP Substrates

**Figure 3:**
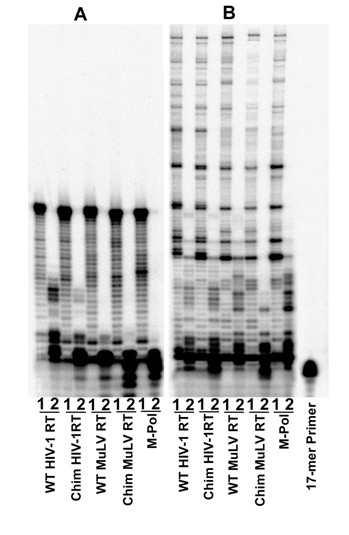
**Use of rNTPs by wild-type RTs and their chimeric derivatives**. The ability of reverse transcriptases from the wild type HIV-1 and MuLV and their chimeric derivatives to incorporate rNTPs was examined on 49-mer U5-PBS DNA (**Panel A**) and U5-PBS-RNA (**Panel B**) templates primed with the 5'-^32^P-labeled 17-mer PBS DNA primer. Reactions were done at 37°C for 30 min as described in Materials and Methods. Lanes 1 and 2 in each panel represent extension reactions done in the presence of 500 μM of dNTPs and rNTPs, respectively.

Since there was a significant change in metal preference for the polymerase function of the chimeric enzymes, it was of interest to examine whether the swapping of the RNase H domains effected any change with respect to substrate discrimination. We therefore examined the ability of the wild-type enzymes and their chimeric derivatives to catalyze the incorporation of rNTPs, using the DNA and RNA templates (Figure 3). The extent of rNTP incorporation with a DNA template by the wild-type HIV-1 RT was greater than that of all other enzymes (Figure 3A). As judged by the band intensity, wild-type HIV-1 RT efficiently incorporated a stretch of several ribonucleotides. In contrast, poor incorporation by the chimeric HIV-1 RT and wild-type MuLV RT was observed. This characteristic of the chimeric HIV-1 RT may be attributed to the presence of the RNase H domain of MuLV RT. In contrast, the rNTP incorporation pattern of M-Pol and chimeric MuLV RT is closely similar to that of the wild-type MuLV RT. Interestingly, all the enzymes except wild-type HIV-1 RT were found to catalyze the cleavage of 3' primer nucleotide in the presence of rNTPs, especially on a DNA template. This may be caused either by pyrophosphorolysis resulting from PPi contamination of the commercial nucleotide preparations or by rNTP-dependent transfer of 3' nucleotide from the primer terminus to rNTP [48]. Cleavage products are abundant in enzymes that are less efficient in rNTP incorporation. With RNA template, wild-type HIV-1 RT is able to incorporate ribonucleotides to a greater extent as compared to that seen with the DNA template (Figure 3B). A similar pattern of rNTP incorporation occurred with the chimeric HIV-1 RT, wild-type MuLV RT, and its pol domain; chimeric MuLV RT exhibited a reduced level of rNTP incorporation

### Fidelity of DNA Synthesis

Since, much like the wild-type MuLV RT, the chimeric HIV-1 RT with the RNase H domain of MuLV RT could discriminate between rNTPs and dNTPs, we examined whether swapping of the RNase H domain influenced the stringency of substrate dNTP selection. We analyzed the pattern of synthesis and extension of the various mispairs by the chimeric enzymes and compared them with those of the wild-type HIV-1 RT and MuLV RT. To determine the pattern of misincorporation at the template position complementary to the missing dNTP, we used the U5-PBS DNA template primed with 5'-^32^P 17-mer PBS primer. For each enzyme, we did four separate reactions in which one of the dNTPs was excluded.

**Figure 4:**
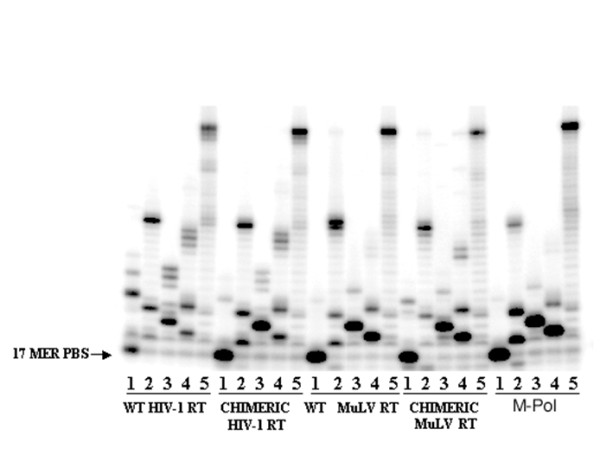
**Fidelity of DNA synthesis by wild-type enzymes and their chimeric derivatives on 49-mer U5-PBS DNA template in the presence of three dNTPs**. The ability of the enzymes to generate and extend mispair in the presence of three dNTPs was assessed on a 49-mer U5-PBS DNA template. The reaction products were analyzed on a denaturing 8% polyacrylamide-urea gel followed by phosphorImager analysis. Lanes 1-4 represent the products formed in the absence of dATP, dCTP, dGTP, and dTTP, respectively. Lane 5 represents the products synthesized in the presence of all four dNTPs. The position of the 17-mer PBS primer is indicated on the left.

In Figure 4, lanes 1-4 represent the reaction conditions in which dATP, dCTP, dGTP, and dTTP were omitted to assess the extent of mispair formation against T, G, C, and A template nucleotides. In all reactions, irrespective of the enzyme, a substantial accumulation of the DNA product occurred at a site before the position of the corresponding missing nucleotide from the reaction mixture. Extension of the misincorporated products into longer products was also evident. However, the extent of mispair extensions differed in case of both RTs and their chimeric derivatives. As shown in Figure 4, HIV-1 RT catalyzes the mispair synthesis and its extension against all the template bases on the DNA template. In contrast, the extent of mispair synthesis and its extension against dT base (see -A lane) catalyzed by the chimeric HIV-1 RT is drastically reduced and similar to MuLV RT, suggesting a possible influence of the RNase H domain of the latter on the polymerase domain of HIV-1 RT. Wild-type MuLV RT characteristically exhibited a significantly higher fidelity than did the wild-type HIV-1 RT. Interestingly, the chimeric HIV-1 RT exhibited higher overall fidelity than did the parental wild-type enzyme, whereas the chimeric MuLV RT had lower fidelity than did the parental wild-type MuLV RT. These results imply that the RNase H domain also contributes to substrate selection and its discrimination. The substrate selection pattern of the M-Pol of MuLV RT was similar to that of the wild-type enzyme, suggesting that the polymerase domain of MuLV RT is a dominant factor in substrate selection.

### Metal Preference for RNase H Activity

**Figure 5:**
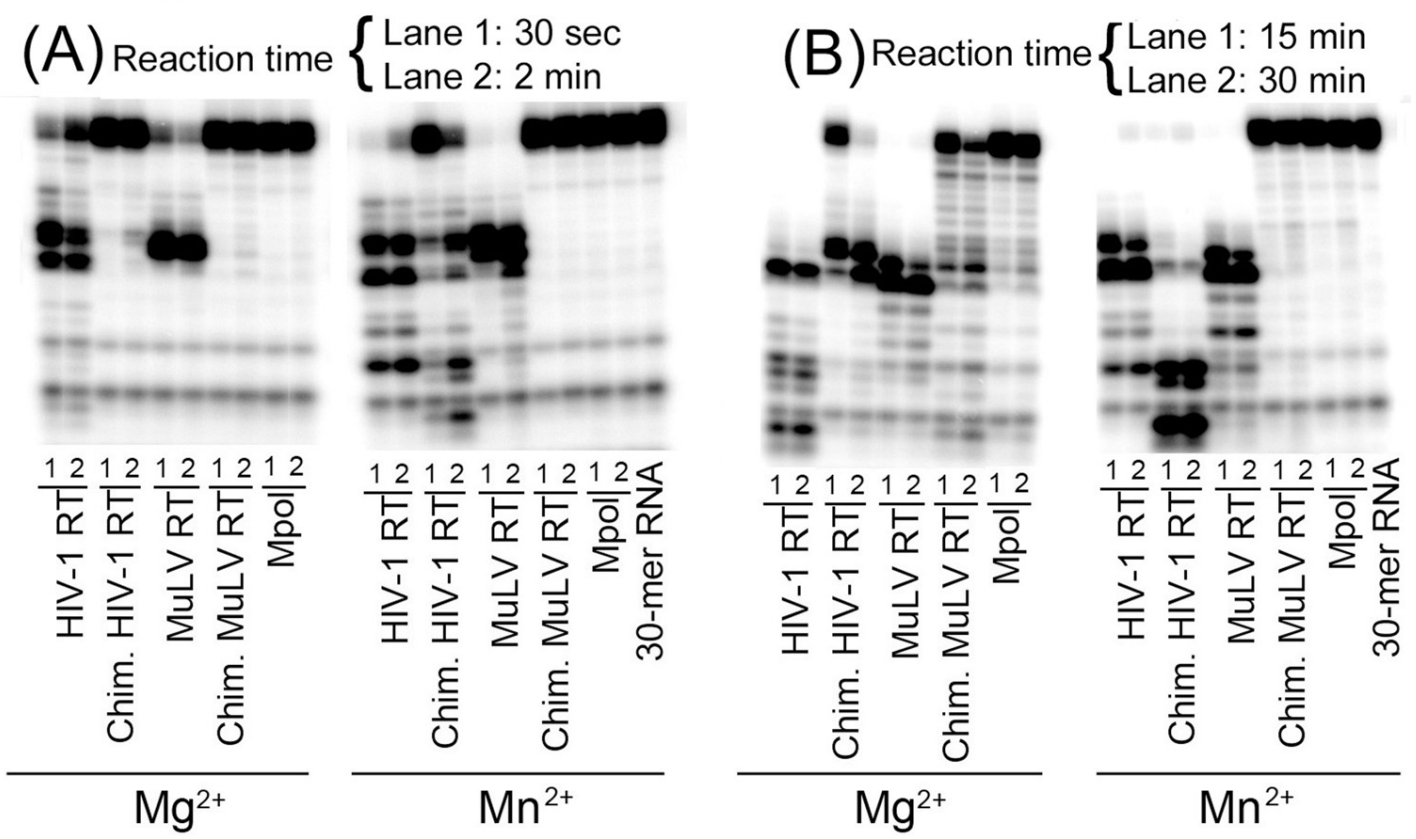
**RNase H activity of wild-type enzymes and their chimeric derivatives**. 5'-^32^P-labeled 30-mer RNA annealed with its complementary 30-mer DNA strand was incubated at 37°C with the wild-type RTs and their chimeric derivatives under standard reaction conditions. The reactions were analyzed on an 8% denaturing polyacrylamide-urea gel. Panels A and B indicate reactions carried out at lower and higher time points, respectively.

Since the metal ion preference for the polymerase activity of the chimeric HIV-1 RT and chimeric MuLV RT is significantly altered due to swapping of the RNase H domains, we examined whether these chimeric enzymes display similar metal preference for RNase H activity. Using a 30-mer RNA-DNA hybrid, we evaluated the cleavage pattern of the 5'-^32^P-RNA strand of the duplex by wild-type enzymes and their chimeric derivatives. As shown in **panel A **of Figure 5, the initial cleavage of the 30-mer RNA strand by the wild-type HIV-1 RT at 30 sec (**lane 1**) and 2 min (**lane 2**) was similar in the presence of either Mg^2+ ^or Mn^2+^, though the processive degradation was highest with Mg^2+^during further incubation (**panel B**) for 15 min **(lane 1) **and 30 min **(lane 2)**. In contrast, chimeric HIV-1 RT exhibited an interesting pattern in response to Mg^2+ ^and Mn^2+^. In the presence of Mg^2+^, initial cleavage of the RNA strand was significantly low (**panel A**). Processive degradation was observed in the presence of Mg^2+ ^only after incubation for 15 min and 30 min (**panel B**), while in the presence of Mn^2+ ^both initial cleavage and progressive degradation could be seen within 30 sec and 2 min, suggesting that this enzyme prefers Mn^2+ ^for its RNase H activity. As expected, MuLV RT displayed a greater preference for Mn^2+ ^for its RNase H activity. Interestingly, chimeric MuLV RT cleaved the RNA strand only in the presence of Mg^2+^; no cleavage activity could be detected with Mn^2+ ^even after prolonged incubation (**panel B**). These results clearly suggest that the metal ion preference for RNase H activity is dictated by the parental RNase H domain.

## Discussion

In the present study we have investigated the role of the RNase H domain of retroviral reverse transcriptase with respect to the substrate selection and metal specificity of their polymerase domains, using HIV-1 RT and MuLV RT as the model enzymes. These enzymes exhibit different polymerase and RNase H activities in response to Mg^2+ ^and Mn^2+^. MuLV RT exhibits approximately 16-fold higher RNase H activity [20] and 10-fold higher polymerase activity on a homopolymeric poly rA template [21] when Mn^2+ ^is used instead of Mg^2+ ^as the divalent cation. In contrast, the polymerase activity of HIV-1 RT is 20- to 50-fold higher in the presence of Mg^2+^[23], while its RNase H activity displays no distinct preference for these metal ions [19]. Interestingly, the fidelity characteristics of DNA synthesis catalyzed by these two enzymes significantly differ, with HIV-1 RT being more prone to make error in DNA synthesis than is MuLV RT. In HIV-1 RT, the catalytic centers of these two domains are separated by approximately 20-21 nucleotides [26]. Specific mutations in the polymerase domain result in the loss of RNase H function, suggesting that these domains, although spatially distinct, are able to communicate with each other. For instance, mutation in the primer grip region in the polymerase domain of HIV-1 RT causes loss of RNase H activity [49,50]. Similarly, a point mutation at position 55 or 156 in the polymerase domain abolishes RNase H activity without significantly affecting polymerase activity [51]. Similarly, expression of the C-terminal RNase H domain of HIV-1 RT resulted in a soluble protein of 15 kD with no detectable enzymatic activity [37,52-54].

Interestingly, the RNase H activity of the 15 kD protein could be restored when that protein was mixed with the polymerase domain of HIV-1 RT (p51 subunit), suggesting a close functional relationship between the two domains [37]. In contrast to HIV-1 RT, the polymerase and RNase H domains of MuLV RT are relatively independent of each other [4,53,55,56]. However, a deletion in the connection subdomain or replacement of the RNase H domain of MuLV RT with the *E. coli *RNase H domain resulted in altered levels of polymerase and RNase H activities, indicating that an interaction between the two domains may exist under physiological conditions [57,58].

To assess how these two domains affect each others' biochemical characteristics, we constructed two chimeric RTs, as described. In-depth biochemical examination of the chimeric HIV-1 RT and MuLV RT has provided evidence that their extrinsic RNase H domain exhibits significant influence on the substrate and metal ion specificity of their native polymerase domain. The chimeric enzymes we constructed, in contrast to an earlier report [53], exhibited both DNA polymerase and RNase H activities. Under our assay conditions, chimeric HIV-1 RT displayed a distinct preference for Mn^2+ ^for polymerase activity on a DNA template (Tables 1 and 2), while its catalytic efficiency on an RNA template with Mn^2+ ^was similar to that of Mg^2+^. In contrast, chimeric MuLV RT retained its distinct preference for Mn^2+ ^on a DNA template, while displaying similar catalytic efficiency with Mn^2+ ^and Mg^2+ ^on an RNA template, suggesting that its pol domain is the dominant factor in metal preference. However, the metal preference of chimeric MuLV RT on an RNA template was significantly altered. As against its parental wild type enzyme, the chimeric MuLV RT manifested similar catalytic efficiency with Mn^2+ ^and Mg^2+ ^on RNA template.

Post *et al*., [57] showed that a chimeric RT construct containing the pol domain from MuLV RT and the RNase H domain from *E. coli *functions in a fashion similar to *E. coli *RNase H, exhibiting nearly 300-fold higher activity with Mg^2+ ^as the divalent cation [57]. However, these authors did not report the influence of *E. coli *RNase H on the metal preference of the chimeric enzyme for polymerase activity. Post *et al*., [57] also demonstrated that after deletion from MuLV RT of the specific region corresponding to the connection subdomain of HIV-1 RT, MuLV RT displayed negligible polymerase activity but retained RNase H activity with Mn^2+^, suggesting the importance of the proper spatial relationship between the two catalytic centers [57].

Although, the chimeric RTs we constructed contained an intact DNA polymerase domain from their wild-type parents, both of them displayed a large increase in their K_m[dNTP] _in the presence of both Mn^2+ ^and Mg^2+^. These chimeric enzymes displayed DNA binding affinities (K_d[DNA]_) similar to those of the wild-type enzyme (Table 3), indicating that the change in the metal preference or the affinity for the substrate is not due to alteration in their DNA binding ability. Thus, the apparent increase in K_m[dNTP] _may be due to changes in the substrate-binding pockets of these enzymes. Further, the relatively lower fidelity of the chimeric MuLV RT and the greater stringency in discrimination of rNTPs versus dNTPs observed with the chimeric HIV-1 RT, especially with a DNA template, indicate that the RNase H domain has a significant effect on the geometry of their substrate binding pockets. It is possible that in the chimeric RTs, the metal coordinating pocket may have acquired distinct conformation with RNA-DNA and DNA-DNA template primer. Alternatively, the metal binding pocket in the chimeric enzymes may have altered as a consequence of global change in their conformation. Taken together, the present studies clearly demonstrate that spatially distinct polymerase and RNase H domains in retroviral RTs communicate and exert significant influence on each other's functions.

## List of abbreviations

U5- PBS RNA template: HIV-1 genomic RNA template corresponding to primer binding sequence (PBS) region; U5-PBS-DNA template; HIV-1 genomic DNA template corresponding to the PBS region; HIV-1 RT: human immunodeficiency virus type 1 reverse transcriptase; MuLV RT: Moloney Murine leukemia virus reverse transcriptase; Poly (rA).(dT)_18_: polyriboadenylic acid annealed with (oligodeoxythymidylic acid)_18_; dNTP: deoxyribonucleoside triphosphate; IMAC: immobilized metal affinity chromatography.

## Competing interests

The authors declare that they have no competing interests.

## Authors' contributions

TTT performed construction and cloning of chimeric RTs, characterization of their polymerase and RNase H activities and wrote the manuscript. AU performed DNA binding studies and determination of metal specificity. VNP conceived the studies, aided in manuscript preparation and participated in experimental design and coordination. All authors read and approved the final manuscript.
